# Extracellular fibrin promotes non-small cell lung cancer progression through integrin β1/PTEN/AKT signaling

**DOI:** 10.1515/biol-2022-0716

**Published:** 2023-09-19

**Authors:** Guilong Li, Jiaying Cai, Jianjun Xie, Yizhi Dai

**Affiliations:** Department of Cardiothoracic Surgery, Zhangzhou Affiliated Hospital of Fujian Medical University, Zhangzhou, Fujian 363000, The People’s Republic of China; Department of Pathology, Zhangzhou Affiliated Hospital of Fujian Medical University, Zhangzhou, Fujian 363000, The People’s Republic of China; Department of Radiotherapy, Zhangzhou Affiliated Hospital of Fujian Medical University, Zhangzhou, Fujian 363000, The People’s Republic of China

**Keywords:** fibrin, integrin β1, PTEN, AKT, NSCLC

## Abstract

The extracellular matrix (ECM) has been strongly correlated with cancer progression in various tumor types. However, the specific mechanisms underlying ECM-associated tumor behaviors remain unclear. In this study, we found an enriched distribution of fibrin in tumor tissues obtained from high-grade non-small cell lung cancer (NSCLC) patients. For further investigation, we established an *in vitro* 3D culture system using fibrin gel and found that NSCLC cells grown in this system exhibited increased stemness and tumorigenesis. Mechanistically, we demonstrated that fibrin facilitated the activation of the phosphatase and tensin homolog (PTEN)/protein kinase B (AKT) signaling pathway through integrin β1. Furthermore, we found that blocking integrin β1 signals enhanced the tumor suppressive effects of chemotherapy, providing a novel approach for clinical therapy for NSCLC.

## Graphical abstract

**Figure:**
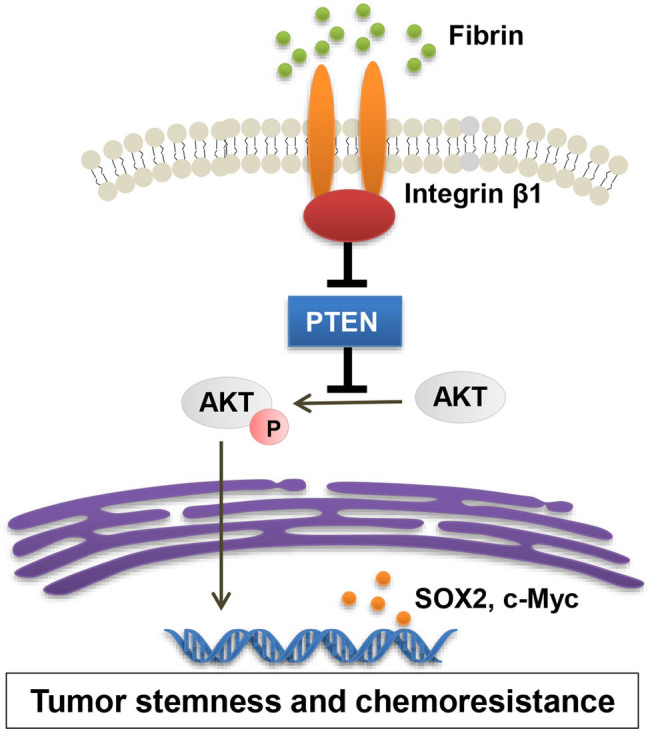

## 1 Introduction

Lung carcinoma is the most common malignant tumor and the leading cause of cancer-associated mortality worldwide [1]. Despite advancements in pre-clinical diagnosis and therapy for localized non-small cell lung cancer (NSCLC), patients still face challenges due to cancer development and distant metastasis driven by NSCLC stem cells [2]. The mechanisms underlying NSCLC progression remain controversial, emphasizing the need to explore these mechanisms and develop innovative approaches to eliminate tumor cells.

Tumor progression involves various biological processes, including oncogene mutations [3], crosstalk between immune cells and cancer cells [4], and extracellular matrix (ECM)-mediated remodeling of tumor stemness [5]. Fibrin, a non-globular fibrous protein associated with blood clotting, is one of the components of the ECM [6,7]. In recent years, increasing evidence has demonstrated the significant involvement of plasma-derived fibrinogen and its activated form, fibrin, in tumor formation, invasion, and metastasis [8,9]. Fibrinogen, a pivotal protein in blood coagulation [10], becomes activated and polymerizes to form fibrin clots following injury. Within the tumor microenvironment, the abundance of vascular tissue frequently leads to the aggregation of fibrinogen or fibrin [11]. Consequently, the presence of fibrin enhances the invasive capacity of tumor cells and offers an ideal avenue for regulating the interplay between tumor cells and their surrounding milieu. Moreover, fibrin is closely associated with tumor angiogenesis, immune evasion, and metastatic processes [12]. Mechanistically, fibrin facilitates the migration and infiltration of tumor cells through various mechanisms, including thrombin activation [13], promotion of angiogenesis [12], and regulation of tumor cell–matrix interactions [14]. Additionally, fibrin regulates the release of cytokines and growth factors within the tumor microenvironment. Fibrin deposition can also create a provisional matrix enriched in growth factors that promote tumor stemness and the capability of cell proliferation [15]. Current studies have provided evidence suggesting that fibrin may participate in the regulation of tumor progression in various tumor types, including lung cancer [16], breast cancer [17], and bladder cancer [18]. Liu et al. reported that fibrin-based 3D gels could promote the proliferation of tumor-repopulating cells in various cancer cell lines, such as melanoma and lung cancer cells [19]. Although enriched fibrin distribution has been observed in various tumor types [20–23], the molecular mechanisms by which fibrin contributes to the progression of NSCLC remain unknown.

Our study found enriched fibrin distribution in tumor tissues obtained from high-grade NSCLC patients. To investigate the effects of fibrin on NSCLC cells, we employed an *in vitro* 3D culture system utilizing fibrin gels. Within these 3D fibrin culture gels, NSCLC cells exhibited enhanced stemness and tumorigenic capabilities. Furthermore, the response of NSCLC cells to fibrin was dependent on the presence of the cell surface integrin β1 receptor, leading to the activation of downstream PTEN/AKT signals and stimulation of tumor growth. Based on these findings, we aimed to investigate the potential of suppressing integrin β1 signaling to enhance the tumor-suppressive effects of chemotherapeutic agents, thereby providing a novel approach for NSCLC therapy.

## 2 Materials and methods

### 2.1 NSCLC cell culture and reagent system

The NSCLC A549 and NCI-H1299 human cells were purchased from the American General Cultural Protection Center and cultured in RPMI-1640 complete media supplemented with 10% fetal bovine serum (Gibco, MA, USA). Fibrin gels were obtained from Sea Run Holdings, Inc. (NJ, USA). Neutralizing antibodies against integrin β1 and integrin β3 were obtained from Selleck Chemicals (NJ, USA). The AKT inhibitor capivasertib was obtained from MedChemExpress (NJ, USA). Cisplatin (Cis) and paclitaxel (PTX) were purchased from Sangon Biotech (Shanghai, China).

### 2.2 Preservation of patient tumor tissue

A total of 22 NSCLC tissues, fixed using formalin and embedded in paraffin, were obtained from the Zhangzhou Affiliated Hospital of Fujian Medical University. The tissue samples were divided into the high-degree (H-D, stage III, *n* = 11) and low-degree (L-D, stages I–II, *n* = 11) groups. All patients were diagnosed with NSCLC and provided written consent to participate in the study. Sample collection and processing adhered to the guidelines outlined in the Helsinki Declaration. This study received approval from the Zhangzhou Affiliated Hospital of Fujian Medical University.


**Informed consent:** Informed consent has been obtained from all individuals included in this study.
**Ethical approval:** The research related to human use has been complied with all the relevant national regulations, institutional policies and in accordance with the tenets of the Helsinki Declaration, and has been approved by Ethics Committee of Zhangzhou Affiliated Hospital of Fujian Medical University.

### 2.3 3D collagen/fibrin culture gel

For the 3D collagen culture system, collagen I (Corning, CA, USA) was diluted to a concentration of 2.5 mg/mL using a cell suspension. To this mixture, 25 μL of PBS and 20 μL of 1 N NaOH were added, resulting in a total volume of 300 μL. The 300 μL collagen mixture was then seeded into a 24-well plate and cultured at 37°C for 90 min. The collagen mixture solidified, after which 1 mL of culture medium was added to the system. After 5 days, the colonies were counted and colony formation was analyzed.

For the 3D fibrin gel culture, fibrin gels were diluted to a concentration of 2 mg/mL using the culture medium. Then, the fibrin gels were further diluted to a concentration of 1 mg/mL with a cell suspension (1,000 cells in a 300 μL mixture). The 300 μL mixture was seeded into a 24-well plate, mixed with 2.5 μL thrombin (0.1 U/mL, Corning, CA, USA), and cultured at 37°C for 90 min. The fibrin mixture solidified, and 1 mL of culture medium was added to the system. After 5 days, the colonies were counted and colony formation was analyzed.

To collect tumor cells from the 3D fibrin or collagen gels, the culture medium was removed from a 24-well plate. Then, 1 mL of Dispase working solution (Corning, CA, USA) was added to the 24-well plate containing the gels and cells. The samples were cultured at 37°C for 10 min, causing the gels to liquefy. Subsequently, samples were added to 5 mL of PBS and centrifuged at 500 *g* for 5 min. The supernatant was removed, and tumor cells were collected for further analysis.

### 2.4 Colony formation analysis

To monitor the 3D colony formation, A549 or NCI-H1299 cells (2,000 cells) were cultured in a 3D fibrin (1 mg/mL) or 3D collagen (2.5 mg/mL) gel and incubated at 37°C for 1 h. Then, 1 mL of culture medium was added to the 24-well plate. After 5 days, the colonies were counted and colony formation was analyzed.

To monitor the 2D flask colony formation, A549 or NCI-H1299 cells (1,000 cells) were cultured in a six-well plate in a complete culture medium. After 10 days, the colonies were counted and colony formation was analyzed.

### 2.5 Cell death and apoptosis analysis

Cell death and apoptosis analysis was conducted using the FITC-Annexin V and PE-PI staining kit (BD, NJ, USA). A549 or NCI-H1299 cells cultured in either 3D or flask formats were collected and treated with the FITC-Annexin V and PE-PI staining solution for 30 min. Then, cell apoptosis was analyzed using a C6 flow cytometer (BD, NJ, USA).

### 2.6 Flow cytometry

The cell pellet was resuspended and washed with PBS supplemented with 2% BSA. Then, the samples were stained with the anti-CD133 primary antibodies (Thermo Fisher, MA, USA) for 20 min at 4°C. The isotype was set as the negative control. Subsequently, the samples were analyzed using a C6 flow cytometer (BD, NJ, USA).

### 2.7 Western blotting

The protein lysates obtained from A549 and NCI-H1299 cells were subjected to SDS–PAGE to separate the proteins, which were then transferred onto polyvinylidene fluoride membranes (Thermo Fisher, MA, USA). The membranes were subsequently incubated with primary antibodies, including anti-integrin β1 (1:1,000; Abcam, Cambridge, UK), anti-PTEN (1:1,000; Abcam, Cambridge, UK), anti-p-AKT (1:500; Abcam, Cambridge, UK), anti-AKT (1:500; Abcam, Cambridge, UK), and anti-β-actin (1:1,000; Abcam, Cambridge, UK). This was followed by treatment with HRP-conjugated secondary antibodies (1:1,000; Abcam, Cambridge, UK).

### 2.8 qPCR analysis

mRNA levels were quantified by real-time PCR using SYBR Green dye (Thermo Fisher, MA, USA). *GAPDH* was used as the reference gene for normalization. The primers used are listed as follows: human *GAPDH* forward primer 5′-GGAGCGAGATCCCTCCAAAAT-3′, reverse primer 5′-GGCTGTTGTCATACTTCTCATGG-3′; human *SOX2* forward primer 5′-GCCGAGTGGAAACTTTTGTCG-3′, reverse primer 5′-GGCAGCGTGTACTTATCCTTCT-3′; human *c-Myc* forward primer 5′-GGCTCCTGGCAAAAGGTCA-3′, reverse primer 5′-CTGCGTAGTTGTGCTGATGT-3′; human *Oct3/4* forward primer 5′-CTGGGTTGATCCTCGGACCT-3′, reverse primer 5′-CCATCGGAGTTGCTCTCCA-3′; human *Klf4* forward primer 5′-CCCACATGAAGCGACTTCCC-3′, reverse primer 5′-CAGGTCCAGGAGATCGTTGAA-3′. Each experiment was repeated independently in triplicate.

### 2.9 Immunohistochemical staining

The NSCLC tissues were fixed in 4% paraformaldehyde for 72 h and then sectioned. The samples were deparaffinized and rehydrated using alcohol and water. For antigen recovery, the samples were treated with sodium citrate buffer at 100℃ for 5 min. For blocking, 5% BSA was used. Subsequently, the samples were incubated overnight at 4℃ with primary antibodies: anti-fibrin α subunit (1:100; Abcam, Cambridge, UK), anti-p-AKT (1:100; Abcam, Cambridge, UK), anti-integrin β1 (1:100; Abcam, Cambridge, UK), and anti-PTEN (1:200; Abcam, Cambridge, UK). Finally, the detection was carried out using the ABC HPR Kit (Thermo Fisher, MA, USA).

### 2.10 Animal experiments

Female NOD-SCID mice, 8 weeks old, were obtained from Huafukang Company (Beijing, China) and raised in a specific pathogen-free room. All animal procedures were approved by the Hospital Animal Care and Use Committee at Zhangzhou Affiliated Hospital of Fujian Medical University. For tumorigenic potential analysis, a total of 1 × 10^5^ A549 or NCI-H1299 cells were subcutaneously implanted into mice. Subsequently, tumors were counted after 30 days (*n* = 10 per group).

For the subcutaneous tumor model, mice were injected with 2 × 10^6^ A549 cells subcutaneously. After 2 weeks, the mice were randomly divided into four groups (*n* = 6 per group). The groups were treated with PBS, integrin β1 neutralizing antibodies (10 μg per mouse, intratumoral injection), Cis (5 mg/kg, intravenous injection), or PTX (20 mg/kg, intravenous injection) every 3 days. Tumor volume was calculated using the formula: Tumor volume = (length × width^2^)/2.


**Ethical approval:** The research related to animal use has been complied with all the relevant national regulations and institutional policies for the care and use of animals, and has been approved by the Ethics Committee of Zhangzhou Affiliated Hospital of Fujian Medical University.

### 2.11 Statistical analysis

Each experiment was performed in triplicate. The data were expressed as the mean ± standard deviation. Statistical analysis was carried out using GraphPad Prism 6.0 (LJ, USA) and SPSS 22.0 software (Chicago, USA). Differences between groups were determined using the analysis of variance or Student’s *t*-test. A *P*-value < 0.05 was considered statistically significant.

## 3 Results

### 3.1 3D fibrin culture promoted NSCLC stemness

**Figure 1:**
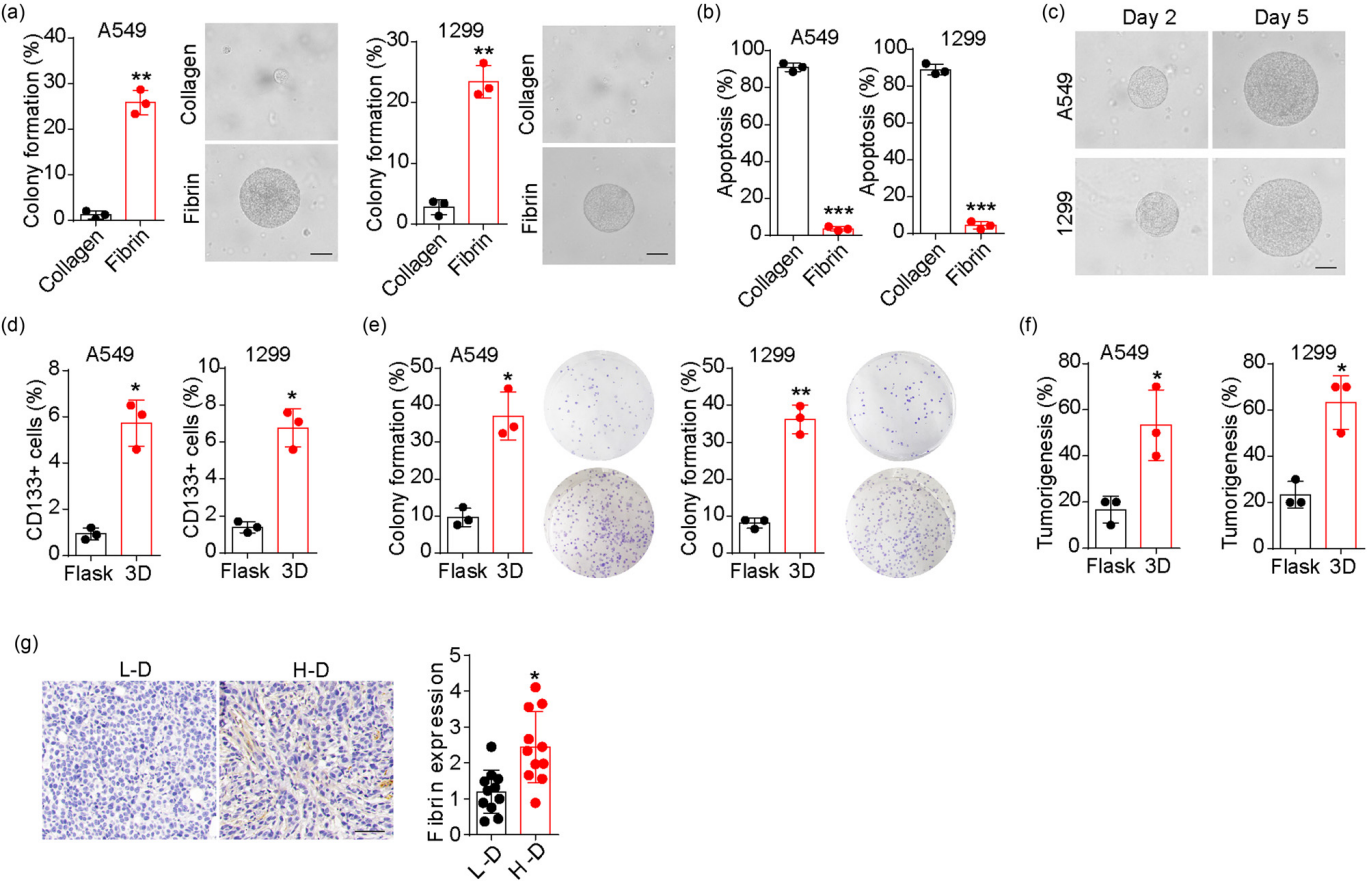
3D fibrin promoted NSCLC stemness. (a) Colony formation rates of A549 and NCI-H1299 cells seeded in 3D fibrin and collagen gels. The colonies were pictured on day 3 and the scale bar is 50 μm. (b) Cells apoptosis of A549 and NCI-H1299 cells seeded in 3D fibrin and collagen gels on day 5. (c) Colonies of A549 and NCI-H1299 cells seeded in 3D fibrin were pictured on days 2 and 5. The scale bar is 50 μm. (d) CD133 positive cells proportion in A549 and NCI-H1299 cells seeded in 3D fibrin on day 5. (e) Colony formation of A549 and NCI-H1299 cells cultured in dish or 3D fibrin gels. (f) Tumorigenesis of A549 and NCI-H1299 cells cultured in dish or 3D fibrin gels. (g) Immunohistochemical staining of fibrin in tumor tissues from high degree (H-D) and low degree (L-D) NSCLC patients (*n* = 11 in each group). The protein level was quantified. The scale bar is 50 μm. * indicates *P* < 0.05. ** indicates *P* < 0.01. *** indicates *P* < 0.001.

Increasing evidence indicates that a 3D culture system utilizing fibrin enhances the stemness of tumor cells in various types of cancer, including melanoma, breast cancer, and hepatoma cells [24,25]. Therefore, we implemented a 3D fibrin gel culture system for NSCLC cell lines (A549 and NCI-H1299) to determine the effects of fibrin on NSCLC progression. Compared to the traditional 3D collagen gel culture system, NSCLC cells cultured in the fibrin gel system exhibited an increased capability of colony formation (Figure 1a) and reduced apoptosis (Figure 1b). Moreover, NSCLC cells in fibrin gels exhibited proliferative characteristics (Figure 1c), whereas cells in collagen exhibited high apoptotic rates (>80%) on day 5. These results indicate that 3D fibrin gel culture facilitates NSCLC cell colony formation. We also examined the expression of CD133, a marker of NSCLC stem cells, in 3D fibrin gel-cultured NSCLC cells. Both A549 and HCI-H1299 cells cultured in 3D fibrin gel showed increased CD133 expression compared to the flask group (Figure 1d). We also evaluated the colony formation capability and tumorigenic potential of these 3D fibrin-cultured NSCLC cells. Consistently, 3D fibrin culture significantly enhanced the colony formation capability of NSCLC cells (Figure 1e). *In vivo* studies demonstrated an enhanced tumorigenic capability in 3D fibrin-cultured A549 and NCI-H1299 cells (Figure 1f), indicating that 3D fibrin gel promotes the stemness of NSCLC cells. Finally, we investigated fibrin distribution in tumor samples obtained from clinical patients. Immunohistochemical staining of the fibrin alpha chain (a component of fibrin) showed elevated expression of fibrin in high-degree (H-D, stage III) tumor tissues compared to the low-degree (L-D, stage I–II) group (Figure 1g). These results suggest that fibrin promotes the tumor stemness of NSCLC cells.

### 3.2 Fibrin facilitates NSCLC stemness through integrin β1 signals

**Figure 2:**
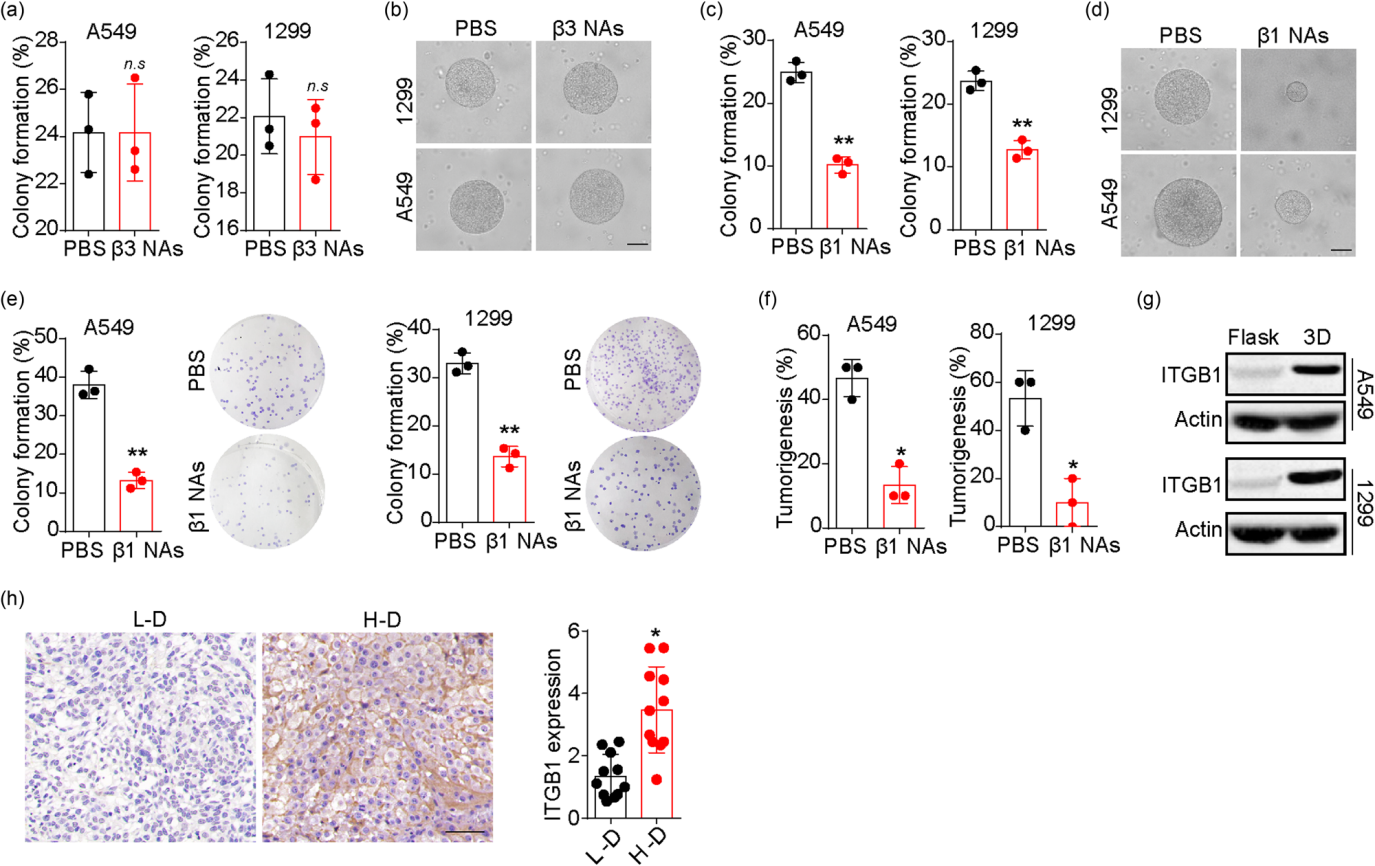
Fibrin facilitated NSCLC stemness through integrin β1. (a) Colony formation rates of 3D fibrin cultured A549 and NCI-H1299 cells treated with PBS and integrin β3 neutralizing antibody (25 μg/mL). (b) Colonies in (a) were pictured on day 3 and the scale bar is 50 μm. (c) Colony formation rates of 3D fibrin cultured A549 and NCI-H1299 cells treated with PBS and integrin β1 neutralizing antibody (25 μg/mL). (d) Colonies in (c) were pictured on day 3 and the scale bar is 50 μm. (e) Colony formation of 3D fibrin gel cultured A549 and NCI-H1299 cells treated with PBS and integrin β1 neutralizing antibody (25 μg/mL). (f) Tumorigenesis of 3D fibrin gel cultured A549 and NCI-H1299 cells treated with PBS and integrin β1 neutralizing antibody (25 μg/mL). (g) Western blotting of integrin β1 in A549 and NCI-H1299 cells cultured in dish or 3D fibrin gels. (h) Immunohistochemical staining of integrin β1 in tumor tissues from high degree (H-D) and low degree (L-D) NSCLC patients (*n* = 11 in each group). The protein level was quantified. The scale bar is 50 μm. * indicates *P* < 0.05. ** indicates *P* < 0.01. n.s. indicates no statistical significance.

The ECM has been shown to play a crucial role in promoting tumor progression by activating integrin-associated signals [26]. Previous studies have identified integrin β3 as a mediator of cellular mechanical signals from the 3D ECM [27]. In this study, we added integrin β3 neutralizing antibodies to the 3D fibrin gel culture system and examined their impact on the colony formation capability of NSCLC cells. However, no significant suppressive effects on colony formation (Figure 2a) or size (Figure 2b) were observed. We then shifted our focus to integrin β1, which has been reported to regulate tumor stemness in colorectal and breast cancer. Interestingly, treatment with integrin β1 neutralizing antibodies significantly suppressed the colony formation (Figure 2c) and growth (Figure 2d) of A549 and NCI-H1299 in 3D fibrin gels. Furthermore, when we collected the integrin β1 neutralizing antibody-treated NSCLC cells in fibrin gels, we observed poor colony formation (Figure 2e) and tumorigenesis (Figure 2f), indicating the crucial role of integrin β1 in NSCLC cells. We also detected elevated expression of integrin β1 in NSCLC cells cultured in 3D fibrin gels (Figure 2g) and in tumor tissues from patients with high-degree NSCLC (Figure 2h). These findings suggest that fibrin promotes NSCLC progression through integrin β1 signals.

### 3.3 3D fibrin promotes tumor progression through PTEN/AKT signals

**Figure 3:**
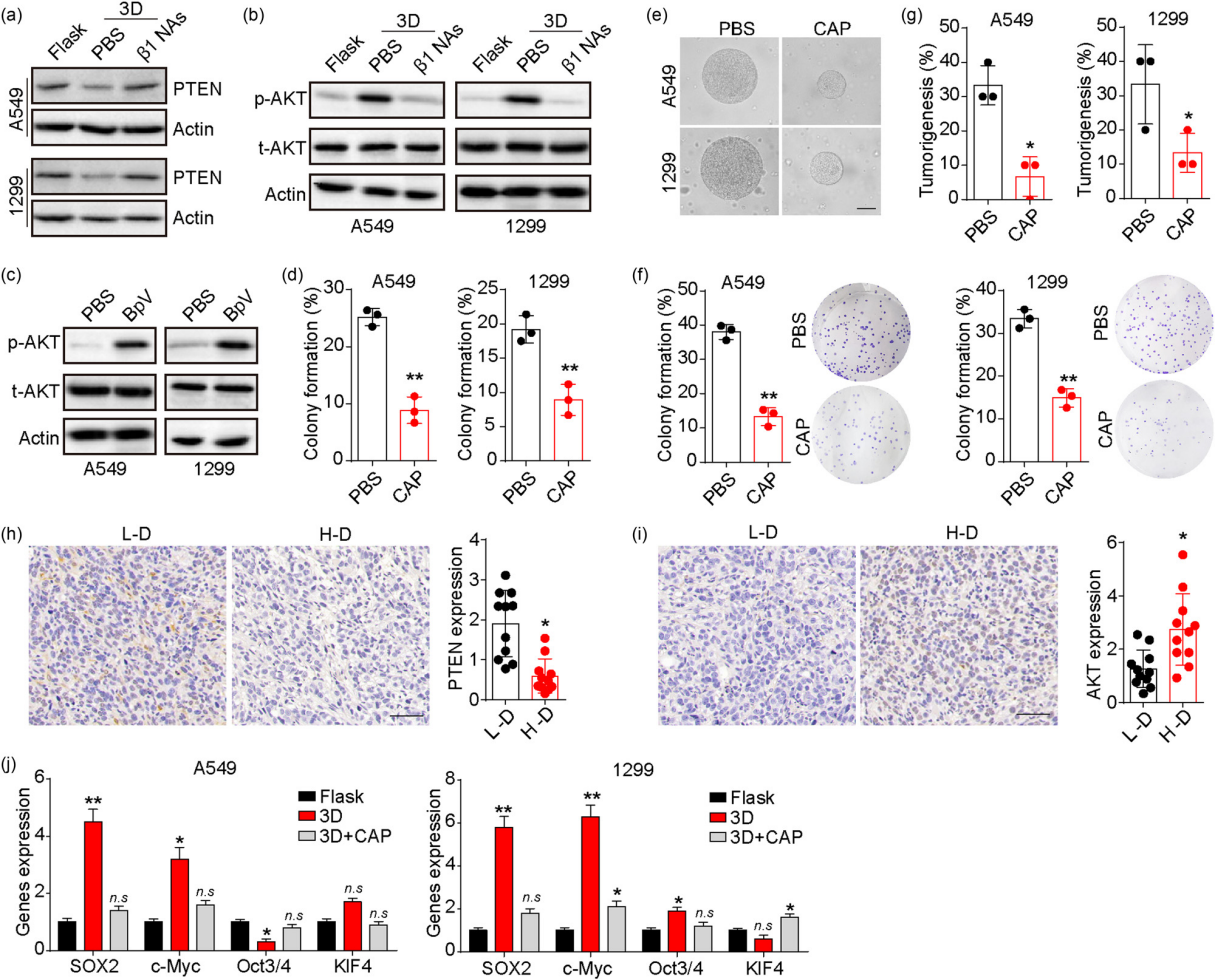
3D fibrin promoted tumor progression through PTEN/AKT signals. (a) Western blotting of PTEN in A549 and NCI-H1299 cells cultured in dish or 3D fibrin gels (treated with PBS or 25 μg/mL integrin β1 neutralizing antibody). (b) Western blotting of phosphorylated/total AKT in A549 and NCI-H1299 cells cultured in dish or 3D fibrin gels (treated with PBS or 25 μg/mL integrin β1 neutralizing antibody). (c) Western blotting of phosphorylated/total AKT in A549 and NCI-H1299 cells treated with PBS or BpV(HOpic) (10 μM). (d) 3D fibrin gel colony formation rate of A549 and NCI-H1299 cells treated with PBS or capivasertib (20 μM). (e) Colonies in (d) were pictured on day 3 and the scale bar is 50 μm. (f) Colony formation rates of 3D fibrin gel cultured A549 and NCI-H1299 cells treated with PBS or capivasertib (20 μM). (g) Tumorigenesis of 3D fibrin gel cultured A549 and NCI-H1299 cells treated with PBS or capivasertib (20 μM). (h) Immunohistochemical staining of PTEN in tumor tissues from high degree (H-D) and low degree (L-D) NSCLC patients (*n* = 11 in each group). The protein level was quantified. The scale bar is 50 μm. (i) Immunohistochemical staining of phosphorylated AKT in tumor tissues from high degree (H-D) and low degree (L-D) NSCLC patients (*n* = 11 in each group). The protein level was quantified. The scale bar is 50 μm. (j) Relative expression of SOX2, c-Myc, c-Kit, Oct3/4, and Klf4 in A549 and NCI-H1299 cells cultured in dish or 3D fibrin gels (treated with PBS or 20 μM capivasertib) using qPCR. * indicates *P* < 0.05. ** indicates *P* < 0.01.

PTEN, a commonly altered tumor suppressor [28], was found to be downregulated in NSCLC cells cultured in our 3D fibrin gels (Figure 3a). Importantly, the suppression of integrin β1 prevented the downregulation of PTEN caused by fibrin gels (Figure 3a), indicating that 3D fibrin gels may regulate NSCLC cells through PTEN-associated signals. We then examined the downstream molecule of PTEN, AKT, in NSCLC cells cultured in the 3D fibrin culture system. Consistently, A549 and NCI-H1299 cells in the 3D fibrin culture system exhibited elevated expression of phosphorylated AKT, which was attenuated by inhibition of integrin β1 (Figure 3b). In addition, treatment of flask-cultured NSCLC cells with the PTEN inhibitor bpV(HOpic) significantly upregulated the expression of phosphorylated AKT (Figure 3c), indicating that 3D fibrin gels stimulated the activation of the PTEN/AKT signaling pathway through integrin β1. To investigate the effect of PTEN/AKT signals on NSCLC, we treated 3D fibrin gel-cultured NSCLC cells with the AKT inhibitor capivasertib. Consequently, capivasertib treatment suppressed colony formation (Figure 3d) and growth (Figure 3e) of NSCLC cells in 3D fibrin gels. Suppression of AKT signaling also inhibited colony formation (Figure 3f) and tumorigenesis (Figure 3g) of NSCLC cells cultured in 3D fibrin gels. Immunohistochemical staining revealed decreased PTEN and increased phosphorylated AKT expression in tumor tissues from patients with high-degree NSCLC (Figure 3h and i), highlighting the crucial role of PTEN/AKT signaling in NSCLC. Furthermore, we examined the expression of pro-survival transcription factors downstream of AKT, including SOX2, c-Myc, c-Kit, Oct3/4, and Klf4. We found increased expression of SOX2 and c-Myc in NSCLC cells cultured in the 3D fibrin culture system, which was reversed by blocking AKT signaling (Figure 3j). These results suggest that 3D fibrin gels promote NSCLC cells through the integrin β1/PTEN/AKT signaling pathway.

### 3.4 Inhibition of integrin signaling improves the anticancer effects of chemotherapeutic agents

**Figure 4:**
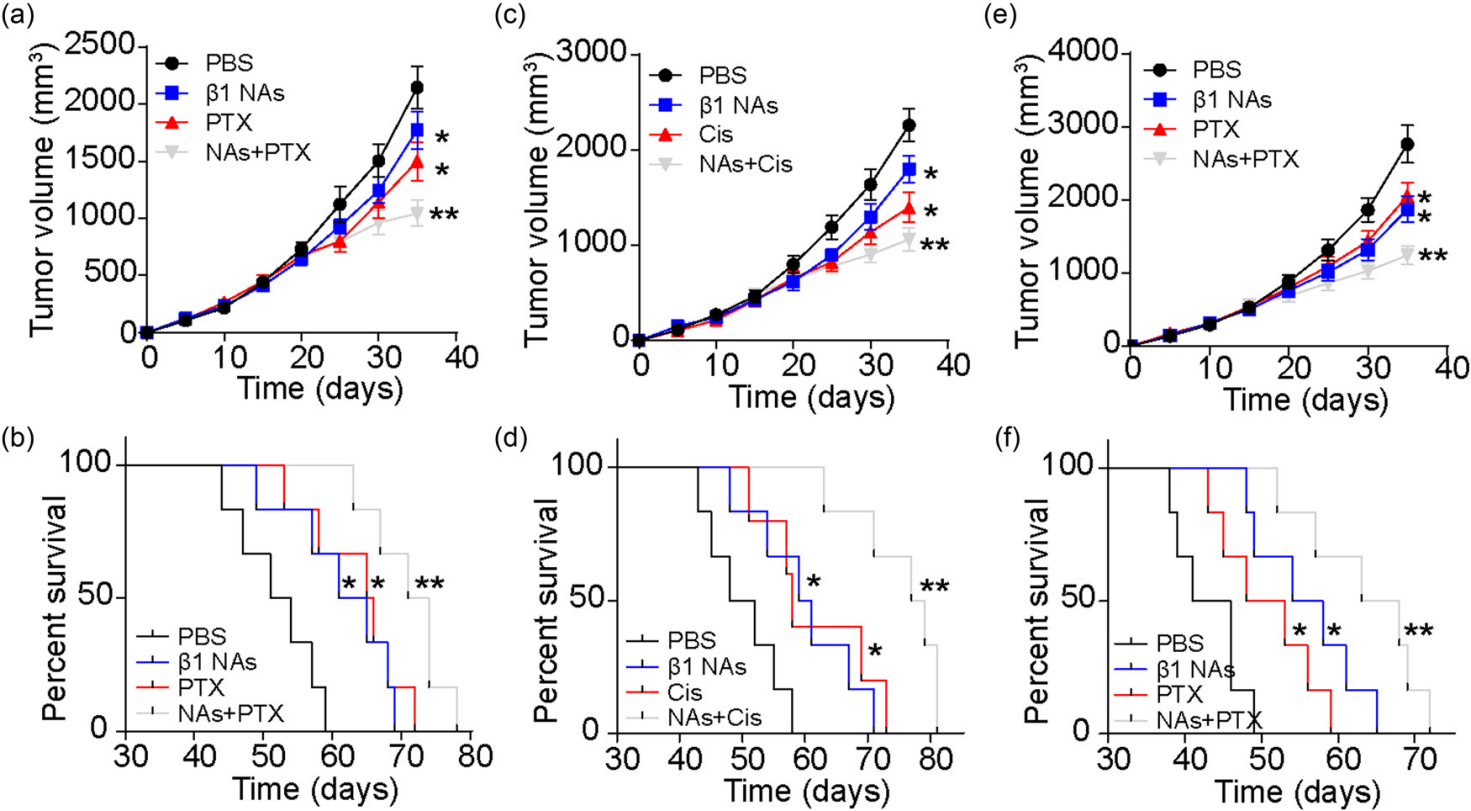
Suppression of integrin signals strengthened anticancer effects of chemotherapy. (a) Tumor volume of A549 bearing mice treated with PBS, integrin β1 neutralizing antibody, PTX, and PTX combined with integrin β1 neutralizing antibody. (b) Survival time of A549 bearing mice treated with PBS, integrin β1 neutralizing antibody, PTX, and PTX combined with integrin β1 neutralizing antibody. (c) Tumor volume of A549 bearing mice treated with PBS, integrin β1 neutralizing antibody, Cis, and Cis combined with integrin β1 neutralizing antibody. (d) Survival time of A549 bearing mice treated with PBS, integrin β1 neutralizing antibody, Cis, and Cis combined with integrin β1 neutralizing antibody. (e) Tumor volume of 3D fibrin gels cultured A549 bearing mice treated with PBS, integrin β1 neutralizing antibody, PTX, and PTX combined with integrin β1 neutralizing antibody. (f) Survival time of 3D fibrin gels cultured A549 bearing mice treated with PBS, integrin β1 neutralizing antibody, PTX, and PTX combined with integrin β1 neutralizing antibody. * indicates *P* < 0.05. ** indicates *P* < 0.01.

Our *in vitro* results have demonstrated that 3D fibrin gels can activate PTEN/AKT signals through integrin β1, indicating that blocking integrin β1 may enhance the efficacy of NSCLC therapy. To investigate this further, we used A549 cells to establish a subcutaneous NSCLC mouse model. Tumor-bearing mice were treated with PBS, integrin β1 neutralizing antibodies, PTX, or a combination of PTX and integrin β1 neutralizing antibodies. The results showed that treatment with integrin β1 neutralizing antibodies, in combination with PTX, effectively inhibited tumor growth and significantly prolonged the overall survival of mice (Figure 4a and b). Similar results were observed with the combination of Cis and integrin β1 neutralizing antibody treatment (Figure 4c and d). To further investigate their clinical relevance in malignant NSCLC, 3D fibrin gel-cultured A549 cells were injected into mice to establish an NSCLC model. Fibrin (1 μg per mouse) was injected into the tumor sites on days 10 and 15 to promote NSCLC progression. Subsequently, the tumor-bearing mice were treated with a combination of integrin β1 neutralizing antibodies and PTX. Interestingly, single PTX treatments showed limited anticancer effects, potentially due to drug resistance induced by fibrin. However, the combination of PTX and integrin β1 neutralizing antibodies effectively inhibited tumor growth and significantly prolonged the survival time of the mice (Figure 4e and f). These results indicate that suppressing integrin β1 signals effectively enhances the tumor-suppressive effects of chemotherapeutic agents, offering a novel therapeutic strategy for NSCLC.

## 4 Discussion

This study provides valuable insight into the role of fibrin in the growth and stemness remodeling of NSCLC cells. Our immunohistochemical analysis of patient samples revealed elevated expression of fibrin in high-degree NSCLC tissues, indicating its potential involvement in tumor progression [29]. Furthermore, we demonstrated that culturing NSCLC cells in 3D fibrin gels significantly enhanced their proliferative capacity and tumorigenic potential compared to 3D collagen gels. Importantly, we proposed that fibrin-induced tumor progression is mediated through the integrin β1/PTEN/AKT signaling pathway. Inhibition of this pathway by blocking integrin β1 or PTEN resulted in suppressed tumor growth and improved outcomes of chemotherapy.

Research has extensively demonstrated the tumor-promoting effects of ECM-derived fibrin. Fibrin plays a significant role in facilitating the migration and infiltration of tumor cells through various mechanisms, including thrombin activation, angiogenesis promotion, and regulation of tumor cell–ECM interactions [12–14]. Furthermore, fibrin acts as a regulator of the release of cytokines [30] and growth factors [11] within the tumor microenvironment. Tumor angiogenesis, a pivotal stage in tumor development, is also influenced by fibrin. Fibrin has been shown to enhance endothelial cell migration and proliferation while modulating angiogenesis-related signaling pathways [31,32]. It is also involved in maintaining the stability of blood vessel structure and regulating vascular permeability. In addition to its effects on angiogenesis, fibrin has been implicated in immune evasion, which is a significant characteristic of tumor progression [12]. Fibrin can regulate tumor cell–immune cell interactions, leading to the suppression of immune responses and fostering tumor escape. Additionally, fibrin has been identified as a modulator of tumor-related inflammatory reactions [33]. Our study further contributes to the understanding of fibrin’s role in governing the stemness of lung cancer cells and sheds light on their resistance mechanisms. Notably, previous studies have suggested that fibrinogen, a homolog of fibrin [34], can bind to integrin αvβ3 and potentiate its ability to stimulate the proliferation of endothelial or tumor cells [35,36]. However, our study revealed that fibrin-induced NSCLC cell growth occurs through an integrin αvβ3-independent pathway [37]. Instead, the activation of pro-survival signals in NSCLC cells requires integrin β1-mediated interaction with fibrin. Suppression of integrin β1 signaling using neutralizing antibodies impaired downstream AKT signaling [38] and reduced tumor growth in mouse models. These findings highlight the potential of targeting integrin β1 as an innovative therapeutic strategy for NSCLC.

PTEN, a dual-specificity protein kinase, plays a crucial role in inhibiting the PI3K/Akt/mTOR signaling pathway by dephosphorylating PI3K [39]. As a significant tumor suppressor gene, PTEN governs cellular processes such as proliferation, apoptosis, and signal transduction. Recently, the relationship between PTEN and tumors has garnered considerable attention within the research community [40]. Genetic alterations such as PTEN loss, mutation, or silencing are frequently observed across various tumor types. These modifications culminated in the functional impairment or complete loss of PTEN, leading to sustained activation of the PI3K/Akt/mTOR signaling pathway, promoting uncontrolled cell proliferation and survival [41]. Consistent with previous investigations, our study confirms that fibrin decreases PTEN expression in lung cancer cells, subsequently activating the AKT signaling pathway. Furthermore, PTEN deficiency impedes apoptotic processes, rendering tumor cells impervious to cell death signals. Notably, our study revealed that decreased PTEN levels contribute to the development of drug resistance in lung cancer. In clinical research, the expression of PTEN, a tumor suppressor, has been closely associated with the prognosis of various cancer types, including breast and prostate cancer, among others. Our study provides evidence that PTEN serves as a tumor suppressor during NSCLC development. In the presence of fibrin, suppression of PTEN expression by integrin β1 leads to the activation of AKT signaling. Decreased PTEN expression and increased AKT expression correlate with the progression of high-degree NSCLC in clinical settings, indicating that PTEN/AKT signaling may serve as an indicator for NSCLC diagnosis or progression analysis.

In summary, our findings demonstrate the critical role of fibrin in NSCLC development through the integrin β1/PTEN/AKT signaling pathway. Targeting integrin β1 may represent an innovative approach to improving outcomes in chemotherapy for NSCLC.
